# Development and evaluation of an innovative model of inter-professional education focused on asthma medication use

**DOI:** 10.1186/1472-6920-14-72

**Published:** 2014-04-07

**Authors:** Sinthia Z Bosnic-Anticevich, Meg Stuart, Judith Mackson, Biljana Cvetkovski, Erica Sainsbury, Carol Armour, Sofia Mavritsakis, Gosia Mendrela, Pippa Travers-Mason, Margaret Williamson

**Affiliations:** 1 Discipline of Pharmacology, Sydney Medical School, University of Sydney, Woolcock Institute of Medical Research, Sydney, Australia; 2 School of Science, Faculty of Health Sciences, Australian Catholic University, Sydney, Australia; 3 New South Wales Ministry of Health, Sydney, Australia; 4 Faculty of Pharmacy, University of Sydney, Sydney, Australia; 5 National Prescribing Service (NPS), Sydney, Australia

## Abstract

**Background:**

Inter-professional learning has been promoted as the solution to many clinical management issues. One such issue is the correct use of asthma inhaler devices. Up to 80% of people with asthma use their inhaler device incorrectly. The implications of this are poor asthma control and quality of life. Correct inhaler technique can be taught, however these educational instructions need to be repeated if correct technique is to be maintained. It is important to maximise the opportunities to deliver this education in primary care. In light of this, it is important to explore how health care providers, in particular pharmacists and general medical practitioners, can work together in delivering inhaler technique education to patients, over time. Therefore, there is a need to develop and evaluate effective inter-professional education, which will address the need to educate patients in the correct use of their inhalers as well as equip health care professionals with skills to engage in collaborative relationships with each other.

**Methods:**

This mixed methods study involves the development and evaluation of three modules of continuing education, Model 1, Model 2 and Model 3. A fourth group, Model 4, acting as a control.

Model 1 consists of face-to-face continuing professional education on asthma inhaler technique, aimed at pharmacists, general medical practitioners and their practice nurses.

Model 2 is an electronic online continuing education module based on Model 1 principles.

Model 3 is also based on asthma inhaler technique education but employs a learning intervention targeting health care professional relationships and is based on sociocultural theory.

This study took the form of a parallel group, repeated measure design. Following the completion of continuing professional education, health care professionals recruited people with asthma and followed them up for 6 months. During this period, inhaler device technique training was delivered and data on patient inhaler technique, clinical and humanistic outcomes were collected. Outcomes related to professional collaborative relationships were also measured.

**Discussion:**

Challenges presented included the requirement of significant financial resources for development of study materials and limited availability of validated tools to measure health care professional collaboration over time.

## Background

Asthma is a chronic respiratory condition. Effective asthma management is dependent on good self-management and optimal use of asthma medications, most commonly delivered via inhaler devices [1,2]. Unfortunately, a high proportion of people with asthma do not use their inhaler device(s) correctly with a majority of patients (between 50 and 80%) demonstrating incorrect technique upon assessment, regardless of the inhaler device used [3-7]. Research in the primary health care setting indicates that educating patients in correct use of their inhalers results is mastery of good inhaler technique and improved asthma control [5,8]. However while patients can master the correct use of their inhalers, the retention of correct technique (technique maintenance) is a challenge, with 50% of patients failing to maintain correct technique over time [7].

It is known that individually, health care professionals (HCPs) play an important role in effectively delivering inhaler technique education to patients [6,9]. However, these educational instructions need to be repeated if correct inhaler technique is to be maintained [4,6,7,10]. Therefore, in clinical practice, it is important to maximise the opportunities to deliver this education across the spectrum of the primary health care setting. In light of this, it is important to explore the way in which pharmacists can work with other HCPs, in particular medical prescribers (general medical practitioners [GPs]) in delivering consistent and repeated inhaler technique education to patients, over time. In addressing this clinical practice problem, there is a need to develop and evaluate effective interprofessional education, which will both i) address the need to educate patients in the correct use of their inhalers as well as ii) equip HCPs with the skills to engage in collaborative relationships with each other. If this can be achieved, issues of medical management, which require long-term and repeated intervention, can be addressed collaboratively.

In considering interprofessional learning (IPL) it is well recognised that just like interprofessional practice, IPL is a complex field of research. Research to date has primarily focused on three aspects: the impact of IPL on i) attainment of new skills and knowledge [11], ii) short-term impact on attitudes towards professional relationship [12], and iii) short-term interprofessional practice [11]. This research has yielded inconsistent findings regarding the effectiveness of IPL; both with regards to interprofessional practice and clinical outcomes for patients [13-18]. Noticeably when it comes to the sustainability of the outcomes over time, few studies have been able to show maintained outcomes following IPL interventions. That is, interventions that have been effective have looked at the initial impact of IPL with most of the evaluative studies using before and after or post intervention research designs [19]. These studies provide little insight into the long-term effects of IPL. Further, methodological issues with IPL interventions have been identified, including: i) lack of a theoretical approach to the design, implementation and evaluation of IPL, ii) minimal addressing of the multilevel barriers to IPL interventions and iii) the practical issues associated with when to introduce IPL and the duration of IPL so that it is feasible.

Fundamentally what is still unknown is whether IPL interventions result in enough individual HCP change in behaviour and attitudes to impact on long-term practice and patient outcomes. This warrants the development of new IPL interventions followed by an evaluation of the IPL intervention on individual HCP change, specifically during the course of learning and over time. Further, the impact on practice and clinical outcomes of patients should also be explored.

Therefore the aim of this study is to:

1. Develop a collaborative care educational module (IPL intervention) for HCPs informed by empirical evidence and a theoretical framework, which focuses on individual transformation in the clinical context of inhaler technique mastery and maintenance.

2. Implement the newly developed collaborative care educational module for HCPs and

3. Compare the newly developed collaborative care educational module for HCPs to standard models of continuing professional education on: the working relationship of HCPs in primary care, the level of collaboration in practice and

4. Evaluate and compare the impact of the educational Models on patient asthma outcomes and inhaler technique of people with asthma.

This project was funded by the Australia Research Council 2008 Linkage Projects Scheme (Project ID LP0882737) and was a research collaboration between the University of Sydney and the National Prescribing Service Limited trading as NPS MedicineWise (A Commonwealth Government of Australia funded independent, evidence-based and not-for-profit organisation, which provides practical tools and information about medicines, health conditions and medical tests to health care providers and consumers).

The study was approved by the University of Sydney Human Research Ethics Committee on the 6^th^ October 2009 (Project Number 10-2009/12200).

## Methods

A mixed methods approach was utilised, commencing with an exploratory qualitative phase in which the key drivers of interprofessional relationships were explored. This empirical data was then considered in light of published research and theoretical frameworks, and used to develop a novel IPL module (described in detail below, under MODEL 3).

Three models of continuing professional education, including the newly developed IPL module, were then implemented and evaluated. This study took the form of a parallel group, repeated measure design, in which HCPs were recruited and allocated to one of four groups (Three interventions (Groups 1, 2 and 3) and one control (Group 4)).

**Figure 1:**
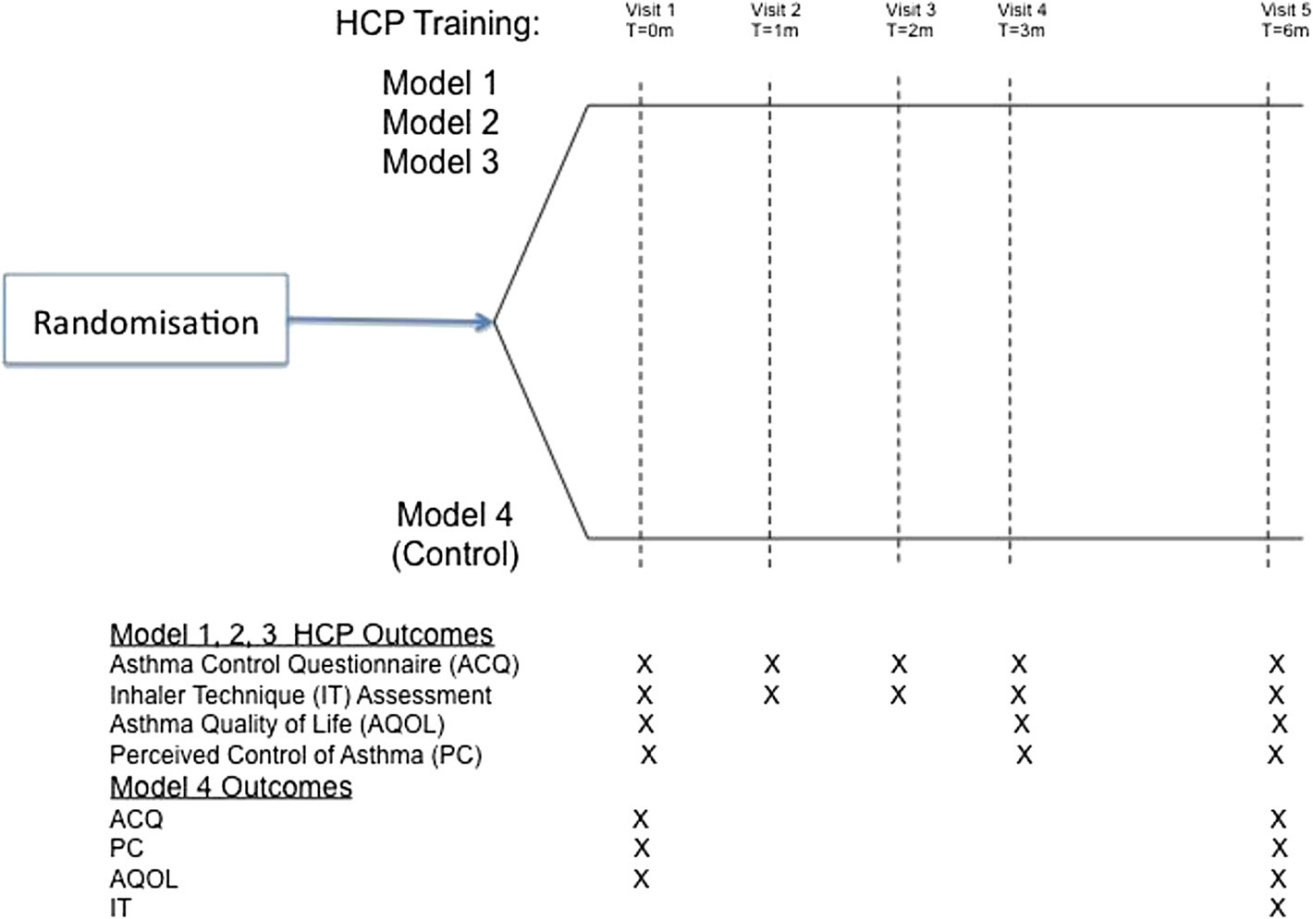
Patient related outcomes.

Based on which one of the three interventions groups the HCPs were allocated to, they received one of three forms of continuing professional education (described below; MODEL 1, MODEL 2, MODEL 3). Following the completion of continuing professional education, HCPs recruited 10 people with asthma into the study and followed them up for a period of 6 months (Figure 1). During the 6 month period, inhaler device technique training was delivered and data on patient inhaler technique, clinical and humanistic outcomes as well as any other regular practices were collected. As the aim of the study was to promote and evaluate the impact of HCP training on collaborative practice around the delivery of the Inhaler technique training, no recommendation regarding which HCP a patient should be visiting for review visits, was made.

Outcomes associated with the relationships between the HCPs and interprofessional practice were evaluated during and immediately following completion of the continuing professional education, over time and at the completion of the study.

### Development of continuing professional education

#### Content

HCPs who were allocated to Groups 1, 2 and 3 all received training on correct asthma inhaler technique (focusing on the Accuhaler®, Turbuhaler®, pressurised Metered Dose Inhaler® and pressurised Metered Dose Inhaler® in combination with a spacer) and how best to educate patients in the correct use of these devices. At the completion of training, HCPs in groups 1, 2 and 3, were skilled in the delivery of an “Inhaler Technique training Service”. This process of training HCPs in the correct use of their inhalers involves a well-articulated process [5].

#### Processes

While the educational content was consistent between Models 1, 2 and 3, the process of the continuing professional education differed.

### MODEL 1 (Standard Model)

The format of delivery for Model 1 was based on the evidence-based continuing professional education module, developed for pharmacists by Basheti et al. [4,5]. This educational module employs a train-the-trainer approach and is of 2.5 hours duration. Model 1 utilised the strategies developed by Basheti et al., [5] and expanded them to incorporate co-training of pharmacists, GPs and their practice nurses i.e. HCPs from all three of the professional groups were brought together and trained in the one continuing professional workshop. HCPs viewed a presentation explaining the theory behind correct inhaler technique, the impact of training on the inhaler technique and asthma outcomes. The importance and potential benefits of HCPs working collaboratively to educate patients in correct inhaler technique use were also discussed. HCPs were shown videos of correct inhaler technique, and then given a resource pack containing placebo devices, a checklist for correct use and time to practise with each other in using these. This model applied one-on-one learning principles whereby HCPs worked in pairs to learn how to use the devices and provide feedback to one another [20]. Facilitators assisted during this process.

### MODEL 2 (Online Model)

Due to the popularity of web-based continuing professional education, a web-based online training module was specifically developed to mimic the educational principles and content of Model 1. Model 2 was supported on the NPS Medicinewise web site and utilised videos and technical animations to train HCPs in asthma device use and in teaching asthma device use. HCPs accessed the module individually at a time convenient to them and were requested to practise independently using the devices supplied in the resource pack.

HCPs using the online module observed both correct inhaler device use, and a model for teaching device use. Learning through observation has been shown to be effective for skills that require spatial sequencing and specific timing [21], both are important elements of correct inhaler device use. Even a single observation of a skill demonstration has been shown to be effective in enhancing performance of that skill [22].

The online module modelled best practice in skills teaching, using a process of instruct – demonstrate – practice, with feedback which focuses on the teaching factors that influence learning of motor skills [23]. Scenario based vignettes showed a HCP and patient interaction in which the functionality and purpose of the inhaler device was explained, the procedure for device use was described (instruct) and then demonstrated by the HCP (demonstrate). The patient was then asked to demonstrate inhaler device use whilst being observed by and receiving feedback from the HCP (practice with feedback). Using the online module, HCP participants were able to observe mock patients making common errors in device use, and were asked to identify the error and suggest remedial action for correction.

### MODEL 3 (Interprofessional Learning Model)

Model 3 was a novel educational intervention for HCPs developed specifically for this study and with particular emphasis on the development of collaborative relationships, along the spectrum of collaborative practice, as described in the Collaborative Working Relationships Model(CWRM) [12,24]. The development of such a collaborative educational intervention, required preliminary explorative research to be conducted. Two clinical settings were chosen in which to explore the nature of existing collaborative relationships as well as identity potential facilitators and barriers to collaborative professional relationships.

The first clinical setting explored was one where there was an existing collaborative relationship in the health care team, oncology teams in a tertiary health care setting. Semi-structured interviews were conducted with oncology pharmacists that had an established role within the team. From the perspective of the oncology pharmacist, an interprofessional approach was seen to be an absolute necessity in terms of optimal patient outcomes. Professional relationships were seen as a critical part of the role of the oncology pharmacists, necessitating a pro-active approach.

The second clinical setting was co-located general practices and pharmacies, representative of the HCP population on which Model 3 would be implemented. Results from their semi-structured interviews and focus groups revealed that co-location is not an essential element to interprofessional relationships between pharmacists and GPs, however, close proximity can facilitate more efficient access. The results from both these interviews helped to identify the current status of professional relationships and how we might proceed to achieve collaboration.

Model 3 mirrored Model 1 in that it took the form of a face-to-face workshop and the content included the theory behind correct inhaler technique, the impact of training and asthma outcomes and the importance and potential benefits of HCPs working collaboratively to educate patients in correct inhaler technique use (*Basheti et al*., [5]). Unlike Model 1, in order to address the process of interprofessional learning, Model 3 was based on the theoretical framework of the sociocultural theory.

**Figure 2:**
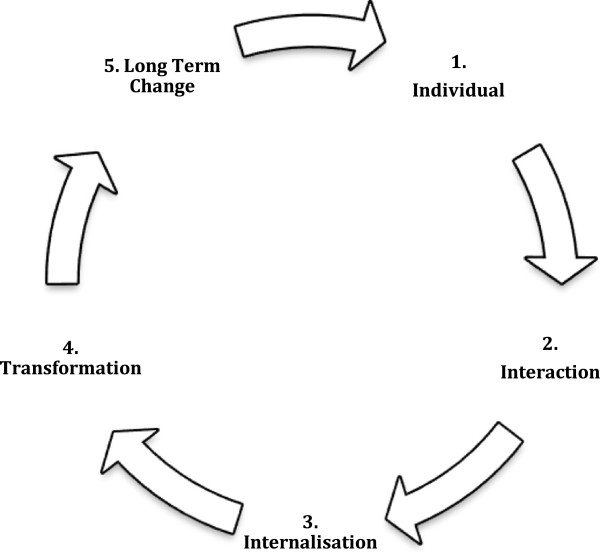
**Socio-cultural change model (Sainsbury) **[27]**.**

Sociocultural theory is an educational framework based on the assumption that humans learn through social interactions. Sociocultural theory is based on the premise that human activities are intrinsically social in nature, and that individual processes and outcomes originate in social practices and interactions [25,26]. The ideals of sociocultural theory are that individuals from different disciplines represent a culture, which influences their social interactions. In this study, we aligned this theory to individual HCPs from different health disciplines interacting in such a way as to influence their working relationship. That is, when individuals interact, they are exposed to information about other cultures, they then internalise this information providing them with the potential to transform their perspectives, attitudes and behaviours towards one another (Figure 2). The sociocultural theory framework can be mapped along five process; i) individual ii) interaction iii) internalisation iv) transformation and v) long term change [27]. Sociocultural theory has not been previously used within the context of collaboration in health care. It was chosen as the theoretical framework for Model 3 (the collaborative training model), based on our preliminary research and other studies [12], showing that GPs and pharmacists exemplify different cultural groups in a professional context.

In order to implement the socio cultural theory framework, the Model 3 workshop was formatted in two parts: Part 1: individual pre-workshop activity completed by participants prior to attending the workshop; Part 2: interactive face-to-face workshop involving all participants

#### Part 1

The pre-workshop activity was developed with the assumption that individual GPs and pharmacists bring unique professional views and biases and that the level of collaboration between these HCP groups is minimal [12,28]. Part 1 was designed to stimulate HCPs to reflect on their own individual perspectives on asthma care using an asthma case study as stimulus material. The case studies presented to the different type of HCPs represented the same patient but from their professional perspective e.g. the patient presenting to the doctors surgery and the patient presenting to the pharmacy. HCPs were required to identify issues, goals and strategies for optimising asthma management from their ‘individual’ perspective.

#### Part 2:

HCPs attended a 2.5 hour face-to-face workshop with 45 minutes allocated to the interactive activity. To optimise the likelihood of future collaboration, GPs and pharmacists who currently or potentially shared patients were paired and seated together around an L-shaped table. HCPs were asked to reflect on the case from Part 1 and share their perspectives. The workshop necessitated HCPs interact and share their unique individual and profession-specific perspectives, as per the pre-workshop activity. Participants were required to identify common and complementary challenges and goals for optimising asthma management, and design strategies for optimising patient care. Although a facilitator was present, their contribution to the discussion was with regards to practical aspects of the workshop such as time keeping.

Group interactions were audio and video taped. Video data was collected as both narrow and wide-angle shots captured by mobile and stationary cameras. Audio data was captured through microphones set up directly in front of participants.

Following the completion of the interactive session, HCPs received training on the correct use of inhaler devices, as in Model 1. In the interests of time, HCPs in Model 3 were required to practise using placebo inhalers in their own time.

### Model 4 - (Control)

HCPs in the control arm received no training prior to the commencement of the study. They were individually visited by a member of the research team and received information on study participation, which for them involved the delivery of their own standard care with the collection of patient data in relation to asthma (details below). At the conclusion of the study, control HCPs were offered access to the online educational material.

### HCP Resources

HCPs in Model 1, 2 and 3 received a resource pack containing: placebo devices (Turbuhaler®, Accuhaler®, pressurised Metered Dose Inhaler® (and Spacer), DVD “Correct Use of Inhaler Devices”, inhaler technique checklist in various formats, participant manual, patient recruitment envelopes (containing Patient ID Card, Patient Information and Consent Forms and Patient Questionnaires). The DVD and inhaler technique checklists were developed by the research team for this study.

HCP participants in the control group (Model 4) were provided with these resources at the conclusion of the study.

### Participant recruitment

#### Health Care Professionals

HCP were recruited through General Practice Networks in the Sydney Metropolitan Region. General Practice Networks are primary health care organisations funded by the Australian Commonwealth Government whose function is to provide medical and allied HCPs within a geographical locality with quality improvement programs. Recruitment through General Practice Networks ensured participating HCPs, and subsequently patients, in each arm of the study would be geographically isolated and thus ensured a reduction in the potential for contamination between the research arms.

The sampling frame for General Practice Networks were those geographically located within two hours drive from central business district of Sydney, Australia. General Practice Networks formally participating in other asthma research activities were excluded from the sampling frame. Seven General Practice Networks with the highest level of asthma prevalence were identified, of which four agreed to participate in the study.

Following enrolment in the study the General Practice Networks facilitated the recruitment of HCPs within their borders. Invitation letters were sent by each General Practice Network Chief Executive Officer to all their general medical practices and pharmacies, inviting all GPs with their practice nurses, and pharmacists to participate in the study. Eligibility criteria for HCPs included:

•Practising within the relevant General Practice Network boundaries,

•Working at the practice/pharmacy a minimum of three days per week,

•Available to participate in the study continuing professional education training (if in Model 1, 2 or 3),

•Willing to recruit 10 patients.

Practice nurses were only eligible if there was a GP from the practice who was participating in the study. Pharmacists were required to have support staff employed within their pharmacy, allowing them to attend to the commitments of the study in a timely manner. GP practices and pharmacies were excluded if they were already participating in another asthma study. HCPs who agreed to participate were required to sign informed consent prior to enrolling in the study.

The four participating General Practice Networks were randomly allocated (using Microsoft Office, Excel®) to Model 1, 2, 3 or 4.

#### Patient recruitment

HCPs recruited patients/participants through their practices/pharmacies. Patients were identified either through patient or medication records or through presentation at the GP practice or pharmacy. Both GPs and pharmacists were able to recruit patients.

Patients were eligible to participate in the study if:

•They were 18 years or older,

•Had a diagnosis of asthma,

•Were currently using an inhaler device for the delivery of asthma preventer medication,

•Had been on the same asthma medication and dose regimen for a minimum of 1 month prior to study enrolment,

•Were willing to complete all visits in the study with participating HCPs.

Patients were excluded if they had Chronic Obstructive Pulmonary Disease, were unable to self-administer their medication, did not speak or understand English, were involved in another asthma clinical trial/study or if they were using Spiriva®.

Patients that met the criteria and agreed to participate were required to sign informed consent prior to their involvement in the study.

### Evaluation

A summary of data collected is outlined in Additional file 1: Table S1.

#### Inter-professional learning (IPL) process evaluation

Qualitative data analysis occurred for both audio and video data which was mapped for processes and content [25,27,28]. IPL process evaluation followed the sociocultural theory framework (Figure 2) and development of specific collaborative behaviours along the CWRM framework [12,24]. In addition, data analysis occurred over two planes: process and content for the individuals and process and content for the group interactions.

#### HCP evaluation

HCP outcomes associated with collaboration and inhaler technique skills were evaluated (Additional file 1: Table S1).

Attitudes towards collaboration: HCPs in Models 1, 2 and 3 were required to complete the Attitudes Towards Health Care Teams Scale (ATHCTS) Questionnaire [29] prior to attending and completing Models 1, 2 and 3 training and immediately after completion of Models 1, 2 and 3 training and at the completion of the study. HCP collaborative process outcomes were measured through the use of the data collection tool (Patient Log described in detail below) as well as semi-structured interviews upon completion of study participation.

HCP inhaler technique assessment: HCPs were assessed on their ability to use placebo inhalers i.e. their inhaler technique. This assessment was based on inhaler technique checklists developed for this study, using published data and manufacturer instructions (see inhaler technique checklists section). For HCPs in Models 1, 2, and 3; inhaler technique of HCPs was assessed within one week of completing Models 1, 2, and 3 training to ensure competency prior to commencing any patient recruitment. HCP inhaler technique assessment was repeated at the conclusion of the study.

### Inhaler technique checklists

#### Accuhaler® technique steps

1. Open inhaler by sliding the thumb grip.

2. Push lever back completely to load dose.

3. Exhale all air out of lungs, away from the inhaler.

4. Keep head upright, lift chin slightly.

5. Hold inhaler horizontally, place mouthpiece between teeth and seal with lips.

6. Inhaler steadily and deeply.

7. Hold breath for as long as comfortable (aim for 10 seconds).

8. Breathe out normally, away from the inhaler.

9. Close inhaler.

#### Metered Dose Inhaler® plus spacer technique steps

##### Single Breath Technique

1. Assemble the spacer (if required).

2. Remove inhaler cap, shake well and insert inhaler into spacer.

3. Exhale all air out of lungs.

4. Keep head upright, lift chin slightly.

5. Place spacer mouthpiece between teeth and seal with lips.

6. Press canister and inhale slowly and deeply from spacer.

7. Hold breath for as long as is comfortable (aim for 10 seconds).

8. Breathe out normally, either into or away from the spacer.

9. Remove spacer from the mouth.

10. Replace the inhaler cap and disassemble the spacer (if required).

#### Metered Dose Inhaler® plus spacer technique steps

##### Multiple Breath Technique

1. Assemble the spacer (if required).

2. Remove inhaler cap, shake well and insert inhaler into spacer.

3. Exhale all air out of lungs.

4. Keep head upright, lift chin slightly.

5. Place spacer mouthpiece between teeth and seal with lips.

6. Breathe in and out of spacer, then press canister.

7. Continue to breathe normally through spacer for a few breaths.

8. Remove spacer from the mouth.

9. Replace the inhaler cap and disassemble the spacer (if required).

#### Metered Dose Inhaler® technique steps

1. Remove inhaler cap and shake well.

2. Exhale all air out of lungs.

3. Keep head upright, lift chin slightly.

4. Place mouthpiece between teeth and seal with lips.

5. Inhale slowly and deeply, pressing canister early.

6. Hold breath for as long as is comfortable (aim for 10 seconds).

7. Breathe out normally, away from the inhaler.

8. Replace inhaler in cap.

#### Turbuhaler® technique steps

1. Unscrew and remove the inhaler cap.

2. Keep inhaler upright.

3. Rotate grip one way, then back, to load dose.

4. Exhale all air out of lungs, away from the inhaler.

5. Keep head upright.

6. Place mouthpiece between teeth and seal with lips.

7. Inhale forcefully and deeply.

8. Pause, then breathe out normally, away from the inhaler.

9. Replace inhaler cap.

HCPs in Model 4 (control) were not required to demonstrate their inhaler technique until the end of the study. At the completion of the study HCPs in Model 4 were given the DVD “Correct Use of Inhaler Devices” and the resource pack. The study researchers assessed the Model 4 HCPs inhaler technique, 1 month after they had received the Resource Pack and been asked to view the DVD.

#### Patient outcomes

During the project, patient outcomes were evaluated at time of recruitment and then 1, 2 3 and 6 months following study commencement (Figure 1). Patient’s asthma control was assessed, using the six question Asthma Control Questionnaire (ACQ) [30,31] and recorded as an Asthma Control Score. Patients were also asked to self complete an assessment consisting of two parts: Part A, the Perceived Control of Asthma (PC) questionnaire [32] and Part B, the Asthma Quality of Life (AQOL) questionnaire [33,34] (Figure 1). These particular questionnaires were chosen, as they are validated tools used for the assessment of clinical and humanistic asthma outcomes; they are commonly used to evaluate asthma outcomes and are sensitive to detect change over time. They have been used to evaluate the impact of inhaler device training in published research [4].

### Electronic data collection

In order to support the practice of sharing patient visits, an electronic, web-based patient log was developed for this study. It allowed for the collection and sharing of patient data through an on-line, patient data collection form (henceforth referred to as the Patient Log). Each patient enrolled in the study had their own unique Patient Log identification number to which the recruiting HCP and another HCP (nominated by the patient) had access during the course of the study. In addition to being used as a data collection tool for the researcher, the Patient Log was a study specific patient record for those HCPs nominated by the patient. It was also a communication tool between the nominated HCPs as at each visit, there was also opportunity for HCPs to include free text comments to each another.

### Sample size

As the ultimate outcome of this research is related to the impact on clinical outcomes, the sample size was based on asthma control. A change in Asthma Control Questionnaire (ACQ) score of 0.5 (i.e. minimal clinical important difference Juniper et al. and Juniper et al.) [30,31] including a completion rate of 60% and a cluster effect of 1.5, it was calculated a total of 71 participants were required in each of Groups 1, 2 and 3 i.e. a total of 234 participants would be required to complete the project.

### Remuneration

GPs and pharmacists were provided with financial remuneration for each completed visit.

Patients were offered a reimbursement (in the form of a shopping voucher) to enroll and complete Visit 1 of the study and received a further shopping voucher to the total value of $AUD50 if they completed all five visits. This was to cover the costs associated with attending visits.

## Discussion

This study aims to address suboptimal asthma management and lack of co-ordinated care in primary care through the development and implementation of a collaborative care module for HCPs.

We aim to address this problem through the development of a new professional continuing education curriculum. The project (facilitated by the academic-industry partnership between the University of Sydney and NPS®) was conducted on a large scale. This unique study is multicentred and evaluates the impact of three distinct forms of HCP continuing professional education. The strength of the novel study is the robust evaluation on the impact of the new educational intervention on HCP relationships, clinical practice and patient asthma outcomes *over time*. The mixed methods design was required to address the research aims as different research questions required different types of data for evaluation. The evaluation tools included video and audio recording of the HCP participating in the collaborative IPL module (Model 3) in addition to the traditional tools of interviews, questionnaires and checklists. The development and use of an electronic data collection tool, Patient Log, was in keeping with current practices and preferences for communication via an electronic medium.

This research poses a number of challenges. Most significant are the financial costs associated with such a large longitudinal study. The financial burden was not only restricted to the participation of health care professionals over a long period of time, but also to the development of multimedia educational resources e.g. the Model 2 Online learning module and the ”Correct Use of Inhaler Devices” DVD. A large proportion of the budgetary requirement was attributed to the development of the online Patient Log. In addition to the financial expense of the Patient Log, its development was further restricted by its necessity to comply with industry requirements and the research framework. As a data collection tool, the Patient Log was a platform that would be unfamiliar to study participants and potentially confusing. Strategies to reduce the impact of this challenge included group and one on one training on the use of the Patient Log as well as the availability of instant support via a telephone hotline.

Further challenges in the study included mirroring the educational content of the Models in three different learning styles. This was strongly challenged in the development of Model 2, the online learning model. Each model has a strong focus on correct inhaler technique, however in Model 2, participants miss out on the interaction and demonstration of correct inhaler technique with fellow participants. To attempt to overcome this, researchers conducted a follow up visit with the participant upon module completion, providing the opportunity for interaction.

The scarcity of validated tools and long term measures for assessing IPL and professional working relationships was an issue in the study. To overcome this, process measures were incorporated in the evaluation, particularly use of Patient Log as a communication tool and the nature of the communication between the HCPs.

The large scale and multicentred requirements of the evaluation pose challenges with regards to recruitment of participants at multiple levels, namely geographically (General Practice Networks), HCPs and subsequently individual patients. The researchers aimed to address this issue by having a broad geographical area in which to recruit General Practice Networks while also accommodating the travel constrictions of the research team, hence improving the likelihood of reaching the required number of consenting participants.

## Abbreviations

HCP/s: Health care professional/s; GPs: General medical practitioners; IPL: Interprofessional learning; ATHCTS: Attitudes towards health care teams scale; ACQ: Asthma control questionnaire; AQOL: Asthma quality of life; PC: Perceived control; NPS: National Prescribing Service.

## Competing interests

The authors declare that they have no competing interests.

## Authors’ contributions

**SZBA** is chief investigator in this study. She has been responsible for all aspects of this research from concept development to implementation. She also wrote and revised all aspects of this manuscript. **MS** is a co-investigator in this research. Her expertise and leadership were fundamental to the development and implementation of MODEL 2. She also provided input into all aspects of study design. She was responsible for overseeing the writing of all the content relating to MODEL 2 in this manuscript and reviewing the whole manuscript. **JM** is a co-investigator in this research. She was the Education and Quality Assurance Program Manager at NPS Medicinewise at the time of the study development. Her expertise was utilised in all aspects of this study design and write up. She has contributed to all aspects of this manuscript by providing feedback. **BC** is the project manager. As a trained researcher, she has contributed input into all aspects of research design. She has been involved in the reviewing and emendation of this manuscript. **ES** is an education researcher with particular expertise in sociocultural theory. She was responsible for overseeing the application of the sociocultural theory into this study. She contributed to the content describing MODEL 3 as well as the outcomes measures used to evaluate “transformation” during the implementation of MODEL 3. **CA** is a co-investigator in this research. She contributed to the design of the qualitative research and the integration of this research throughout MODEL 3. She also actively contributed to the conceptualisation of the educational context regarding inhaler technique training. She reviewed all aspects of this manuscript and provided valuable feedback to content and style. **SM** was responsible for designing the details of MODEL 3 and was involved in the writing of this aspect of the manuscript. **GM** is a technology and on-line education expert and was responsible for developing the content of MODEL 2. She contributed to the content relating to MODEL 2 in this manuscript. **PTM** was responsible for producing the educational videos included in MODELS 1 and 3 and presented in MODEL 2. She provided content relating to the education videos within this manuscript. **MW** is a co-investigator in this research. She is the Research Director at the NPS Medicinewise and as an experienced researcher has been involved in all aspects of this study design and write up. She has contributed to all aspects of this manuscript by providing feedback during the early drafts all the way through to the final drafts. All authors read and approved the final manuscript.

## Pre-publication history

The pre-publication history for this paper can be accessed here:

http://www.biomedcentral.com/1472-6920/14/72/prepub

## Supplementary Material

Additional file 1: Table S1 Outcome Measures and collection points. Click here for file
