# Supplementary material for: Development and evaluation of an innovative model of inter-professional education focused on asthma medication use
Source: BMC Med Educ. 2014 Apr 7;14:72. doi: 10.1186/1472-6920-14-72 (PMC4234384; doi:10.1186/1472-6920-14-72)
Supplement: Additional file 1: Table S1 — Outcome Measures and collection points. [file 1472-6920-14-72-S1.docx]

**Additional file 1: Table S1: Outcome Measures and collection points**

VISIT 1 2 3 4 5

|  | **Time (months)** | **-2** | **0** | **1** | **2** | **3** | **6** |
| --- | --- | --- | --- | --- | --- | --- | --- |
| **Level of measurement** | Indicator |  |  |  |  |  |  |
|  |  |  |  |  |  |  |  |
| **HCP**  **RELATIONSHIP** | ATHCTs (Attitudes towards health care teams scale) | ✓ | ✓ |  |  |  | ✓ |
|  | Qualitative feedback group |  |  |  |  |  | ✓ |
|  |  |  |  |  |  |  |  |
| **PATIENT** | Inhaler technique |  | ✓ | ✓ | ✓ | ✓ | ✓ |
| **CLINICAL** | Asthma control |  | ✓ | ✓ | ✓ | ✓ | ✓ |
|  | Asthma medication profile |  | ✓ | ✓ | ✓ | ✓ | ✓ |
|  | Asthma History/Severity |  | ✓ |  |  |  | ✓ |
|  | ***Note for above :***  Intervention patients will be assessed at 0,1,2,3,6 months  control patients will be assessed at 0,6 months |  |  |  |  |  |  |
| **PATIENT** | Asthma quality of life |  | ✓ |  |  | ✓ | ✓ |
| **HUMANISTIC** | Asthma perceived control |  | ✓ |  |  | ✓ | ✓ |
|  | Patient satisfaction |  |  |  |  | ✓ | ✓ |
|  | Pharmacist satisfaction |  |  |  |  | ✓ | ✓ |
|  | GP satisfaction |  |  |  |  | ✓ | ✓ |
|  | ***Note for above :***  Model 1, 2 and 3 patients will be assessed at 0,3,6 months; control patients will be assessed at 0,6 months |  |  |  |  |  |  |
| **PROCESS** | Number of GP practices recruited | ✓ |  |  |  |  |  |
| **MEASURES** | Number of pharmacies recruited | ✓ |  |  |  |  |  |
|  | Number of GPs recruited | ✓ |  |  |  |  |  |
|  | Number of practice nurses recruited | ✓ |  |  |  |  |  |
|  | Number of pharmacies recruited | ✓ |  |  |  |  |  |
|  | Number of participant attending workshops | ✓ |  |  |  |  |  |
|  | Pattern of health professional attendance: GP practice and pharmacy | ✓ | ✓ | ✓ | ✓ | ✓ | ✓ |
|  | Nature of interaction between GP practice and pharmacy | ✓ | ✓ | ✓ | ✓ | ✓ | ✓ |
|  | Barriers and facilitators to implementation |  | ✓ |  |  |  | ✓ |
|  | Recruitment rate |  | ✓ |  |  |  | ✓ |
|  | Retention rate of health professional participants |  |  |  |  |  | ✓ |
|  | Sustainability of service delivery (time) |  |  |  |  |  | ✓ |
